# Voxelwise meta-analysis of gray matter anomalies in progressive supranuclear palsy and Parkinson's disease using anatomic likelihood estimation

**DOI:** 10.3389/fnhum.2014.00063

**Published:** 2014-02-18

**Authors:** Na Shao, Jing Yang, Jianpeng Li, Hui-Fang Shang

**Affiliations:** Department of Neurology, West China Hospital, Sichuan UniversityChengdu, China

**Keywords:** progressive supranuclear palsy, Parkinson's disease, subtraction meta-analysis, anatomic likelihood estimation, voxel-based morphometry, gray matter volume

## Abstract

Numerous voxel-based morphometry (VBM) studies on gray matter (GM) of patients with progressive supranuclear palsy (PSP) and Parkinson's disease (PD) have been conducted separately. Identifying the different neuroanatomical changes in GM resulting from PSP and PD through meta-analysis will aid the differential diagnosis of PSP and PD. In this study, a systematic review of VBM studies of patients with PSP and PD relative to healthy control (HC) in the Embase and PubMed databases from January 1995 to April 2013 was conducted. The anatomical distribution of the coordinates of GM differences was meta-analyzed using anatomical likelihood estimation. Separate maps of GM changes were constructed and subtraction meta-analysis was performed to explore the differences in GM abnormalities between PSP and PD. Nine PSP studies and 24 PD studies were included. GM reductions were present in the bilateral thalamus, basal ganglia, midbrain, insular cortex and inferior frontal gyrus, and left precentral gyrus and anterior cingulate gyrus in PSP. Atrophy of GM was concentrated in the bilateral middle and inferior frontal gyrus, precuneus, left precentral gyrus, middle temporal gyrus, right superior parietal lobule, and right cuneus in PD. Subtraction meta-analysis indicated that GM volume was lesser in the bilateral midbrain, thalamus, and insula in PSP compared with that in PD. Our meta-analysis indicated that PSP and PD shared a similar distribution of neuroanatomical changes in the frontal lobe, including inferior frontal gyrus and precentral gyrus, and that atrophy of the midbrain, thalamus, and insula are neuroanatomical markers for differentiating PSP from PD.

## Introduction

Progressive supranuclear palsy (PSP) is an atypical Parkinsonism neurodegenerative disease that often affects adults between 60 and 65 years of age (Armstrong, 2007). PSP is characterized by progressive vertical gaze palsy, early postural instability with falls, late frontal cognitive dysfunction, and poor response to levodopa (Barsottini et al., 2010; Hirano et al., 2010; Stamelou et al., 2010). Recent studies have suggested that the morbidity of PSP may be higher than is usually considered (Nath et al., 2001). Parkinson's disease (PD), the second most common neurodegenerative disease, is characterized by rigidity, tremor, bradykinesia, and posture instability (Tanner and Goldman, 1996). Although symptoms such as vertical gaze palsy and early falls are specific to PSP, differential diagnosis from PD can still be difficult at early stages (Nilsson et al., 2007; Varrone et al., 2007).

Structural magnetic resonance imaging (MRI), functional MRI (fMRI), and positron emission tomography (PET) are increasingly being used in the differential diagnosis of PD and related disorders (Juh et al., 2004). Voxel-based morphometry (VBM), a time-efficient and automated tool, can reveal morphological changes throughout the whole brain on a voxel-wise basis (Ashburner and Friston, 2000). VBM has been widely used in studies on PSP and PD (Brenneis et al., 2004; Burton et al., 2004; Beyer et al., 2007; Agosta et al., 2010). However, the results of VBM studies are controversial or not completely consistent because of small and heterogeneous samples of participants and substantial methodological differences among studies. For example, some researchers (Agosta et al., 2010; Lehericy et al., 2010) have reported cerebellum atrophy in PSP, whereas others (Brenneis et al., 2004; Boxer et al., 2006) did not find such abnormalities in PSP. Similarly, some articles (Focke et al., 2011; Fernández-Seara et al., 2012) have reported precuneus impairment in PD, whereas others (Dalaker et al., 2010; Hong et al., 2012) failed to replicate such findings in PD. Thus, the application of meta-analysis to identify consistent results of VBM studies on PSP and PD has gained increasing interest. We published a VBM meta-analysis on PD in 2012, which included articles from 1995 to 25 October 2010 (Pan et al., 2012). As more VBM studies on PD published since then, we updated the results of meta-analysis on PD to explore more accurate GM changes in PD.

The anatomic likelihood estimate (ALE) method, a newly developed statistical technique, is a powerful voxel-based meta-analytic technique originally designed for functional neuroimaging studies (Turkeltaub et al., 2002; Laird et al., 2005). However, ALE has been effectively applied to anatomical image datasets, such as VBM and diffusion tensor imaging studies (Ellison-Wright et al., 2008; Ellison-Wright and Bullmore, 2009). Identifying consistent structural abnormalities may provide insight into the pathophysiology of PSP and PD. PSP can be easily misdiagnosed as PD. Given that the midbrain and thalamus are reportedly more severely damaged in PSP, and that the impairment of these two areas contributes to classical symptoms, such as vertical gaze palsy (Kato et al., 2003) and postural instability (Zwergal et al., 2011), we speculated that atrophy of the midbrain and thalamus may be a neuroanatomical marker for the differentiation of PSP from PD. Therefore, subtracting the results of meta-analysis of PSP and PD will reveal the difference in pattern change between PD and PSP, thus confirming our abovementioned hypothesis and increasing diagnostic accuracy in clinical practice.

This study aimed to identify the consistent finding of gray matter volume (GMV) changes in PSP and PD separately using the ALE approach (Turkeltaub et al., 2002; Laird et al., 2005). Contrast analysis was performed through ALE subtraction analysis (Eickhoff et al., 2011) to identify the differential pattern of GMV changes between PSP and PD.

## Materials and methods

### Data sources and study selection

Systematic and comprehensive searches were conducted on the Embase and PubMed databases from January 1995 to April 2013 using the following key words: (“voxel,” “voxel-based,” “voxel-wise,” “morphometry,” “VBM,”) and (“PSP,” “Steele–Richardson–Olszewski syndrome,” “ophthalmoplegia,” “Parkinson,” “Parkinsonism,” “Paralysis Agitans,” and “PD”). The reference lists of relevant papers were also searched for additional studies.

Studies were included if they met the following criteria: (1) reported on VBM as applied to GM density (GMD) or GMV changes from a comparison of PSP and healthy control (HC), or comparison of PD and HC (PD-HC), or comparison of PSP and PD (PSP-PD); (2) reported whole-brain GM changes in standard Talairach or Montreal Neurological Institute (MNI) stereotactic spatial coordinates; (3) used significance thresholds that are either corrected for multiple comparisons or uncorrected but with spatial extent thresholds; (4) were published in English with peer review. For studies that met the aforementioned inclusion criteria and were performed by the same authors or research institution, only the study with the largest sample size was selected.

Studies were excluded if they suffered from at least one of the following deficiencies: (1) sufficient data could not be obtained even after corresponding with author/s by email or phone; (2) the data overlapped with those of another article.

The method used in the current study was based on the guidelines of “Meta-analysis of Observational Studies in Epidemiology” for the meta-analysis of observational studies (Stroup et al., 2000).

**Figure 1:**
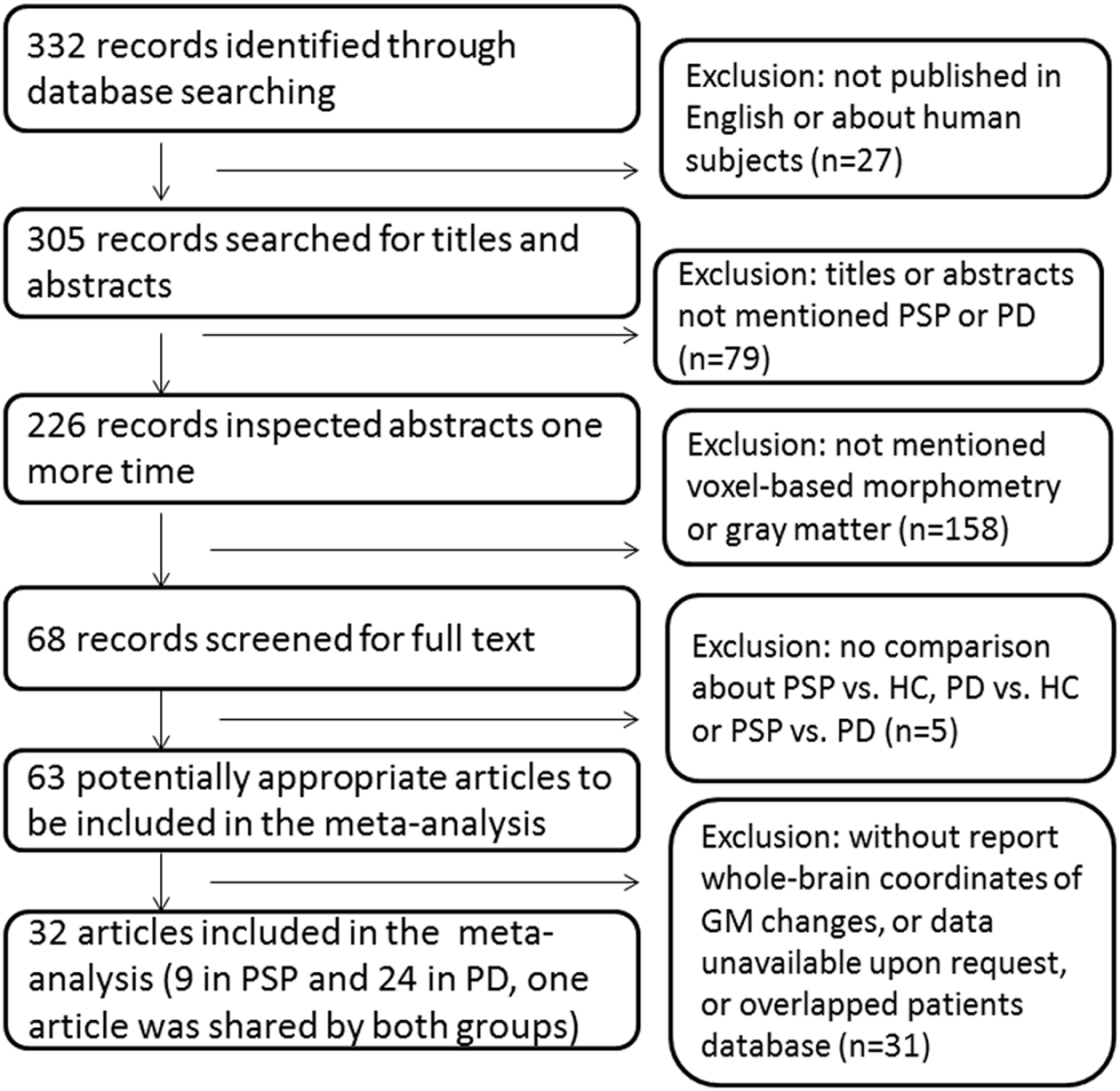
**Flow-chart of searching strategy**.

Study selection was performed by one author (Na Shao) and verified by another author (Jing Yang) independently. The process of study selection was provided in Figure 1.

### Data extraction

Ginger ALE (http://brainmap.org/ale/index.html) was used to transform reported coordinates into the stereotactic space of Talairach. Reported coordinates in a stereotactic space of the MNI were converted to Talairach coordinates using Lancaster transform (Lancaster et al., 2007). Reported coordinates transformed to Talairach space using the Brett transformation (Brett et al., 2002) were converted back to MNI space and then reconverted to Talairach also using the Lancaster formula (Lancaster et al., 2007). Two authors (Na Shao and Jing Yang) performed data extraction independently.

### Meta-analysis of VBM studies

Meta-analysis was performed using Ginger ALE software 2.1.1 (http://www.brainmap.org) and the Talairach stereotactic coordinates derived from the included articles (Eickhoff et al., 2012). The reported coordinates were modeled using a 3D Gaussian distribution defined by full width at half maximum (FWHM) (Laird et al., 2005). The FWHM was set according to a quantitative uncertainty model (Eickhoff et al., 2009). Separate ALE maps were created for multiple comparisons associated with PSP–HC and PD–HC. Afterward, in order to identify the differential pattern of GMV changes between PSP and PD, ALE subtraction analysis (Eickhoff et al., 2011) evaluating the differences between the ALE maps of PSP–HC and PD–HC was carried out, taking into account the potential difference in the sample size. These maps were thresholded at *p* < 0.05 corrected for multiple comparisons using False Discovery Rate (Benjamini and Hochberg, 1995). An extent-threshold of 100 mm^3^ was applied. The coordinates of the weighted center were generated for each cluster. The resulting significant anatomical areas were labeled by referring to probabilistic cytoarchitectonic maps of the human brain using the SPM Anatomy Toolbox v1.8 (Eickhoff et al., 2005). This labeling presented the attribution of each voxel of the reference space to the most likely cytoarchitectonic area (Eickhoff et al., 2005). Rest software was used to visualize the ALE maps overlaid onto an average brain template MNI/ICBM 152 (http://www.bic.mni.mcgill.ca/ServicesAtlases/ICBM152NLin2009).

## Results

### PSP < HC

**Figure 2:**
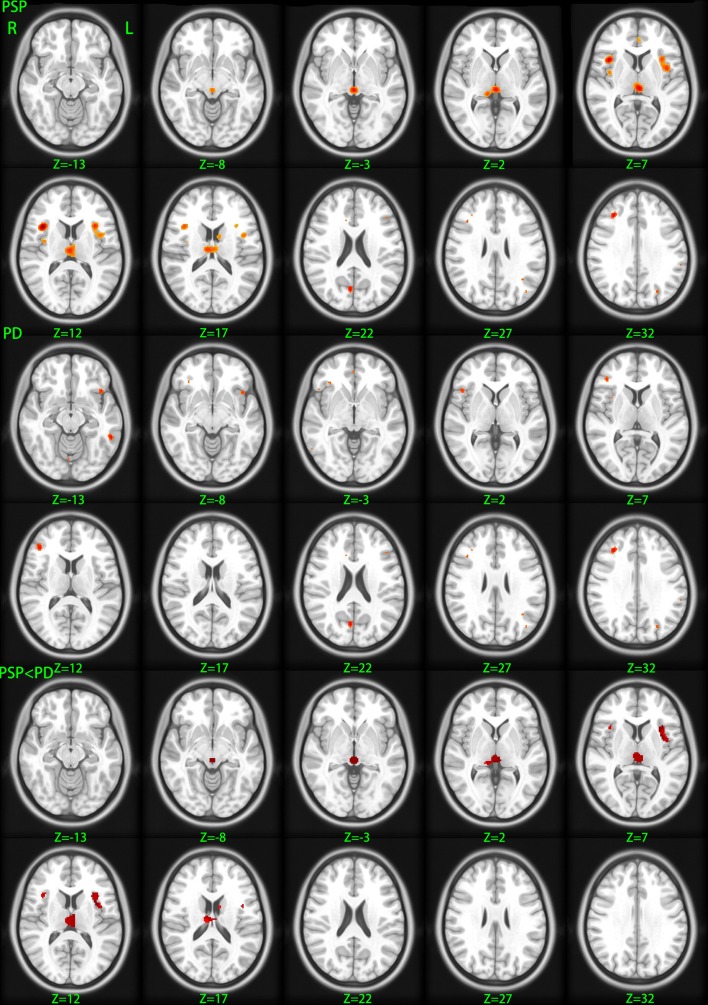
**Gray matter volume changes betweeen different groups**. PSP, gray matter decreases in patients with progressive supranuclear palsy relative to healthy control; PD, gray matter decreases in patients with Parkinson's disease relative to healthy control; PSP < PD, diminished gray matter volume in progressive supranuclear palsy patients relative to Parkinson's disease patients. Significance thresholded with a false discovery rate at *P* < 0.05 and a cluster extent threshold of 100 voxels; z, z coordinates in Talairach space; L, left; R, right.

We identified 32 studies in the database searches (Figure 1) including nine PSP-HC articles (Brenneis et al., 2004; Cordato et al., 2005; Boxer et al., 2006; Padovani et al., 2006; Agosta et al., 2010; Lehericy et al., 2010; Takahashi et al., 2011; Ghosh et al., 2012; Giordano et al., 2013). One hundred and fourty-five PSP patients and 217 HC were included in this study and a total of 104 foci with reduced GMV were reported. The main characteristics of the included participants are presented in Table 1. Thirty PSP patients from two studies had mild cognitive impairment (Padovani et al., 2006; Takahashi et al., 2011). GM reduction was observed in the bilateral thalamus, midbrain, insula, basal ganglia, inferior frontal gyrus, left precentral gyrus, and the left anterior cingulate by using the ALE approach (Figure 2). No increased GMV relative to HC was observed in the brain regions of PSP patients. Similar findings were revealed after excluding two studies conducted on PSP patients with mild cognitive impairment (Padovani et al., 2006; Takahashi et al., 2011) (Supplemental Figure 1).

**Table 1:** **Summary of PSP articles included in the meta-analysis**.

**Study**	**Number of subjects (*F*)**	**UPDRS-III**	**MMSE (*SD*)**	**Age (*SD*)**	**Significance threshold**	**Diagnostic criteria**	**Diagnosis**	**Scanner (*T*)**	**Thickness (mm)**	**FWHM (mm)**	**# Foci**
Agosta et al., 2010	PSP: 20(6)	32.8	27.0 (NA)	64.9 (NA)	*P* < 0.001	NINDS-SPSP	Probable PSP: 18	1.5	2.5	8	16
	HC: 24(11)	–	NA	63.8 (NA)	(uncorrected)		Probable PSP: 2				
Boxer et al., 2006	PSP: 15(6)	NA	24.0 (3.2)	70.9 (6.9)	*P* < 0.05	NINDS-SPSP	Probable PSP: 15	1.5	NA	12	6
	HC: 80(43)	–	NA	67.9 (8.6)	(FWE)						
Brenneis et al., 2004	PSP: 12(NA)	38.9	NA	67.5 (6.6)	*P* < 0.05	NINDS-SPSP	Probable PSP: 12	1.5	1.5	8	12
	HC: 12(NA)	–	NA	60 (5.8)	(unclear)						
Ghosh et al., 2012	PSP: 22(NA)	33.8	NA	71.1 (8.6)	*P* < 0.05	NINDS-SPSP	Definite PSP: 9	3	1.25	16	19
	HC: 20(NA)	–	NA	71.4 (7.6)	(FWE)		NA: 13				
Lehericy et al., 2010	PSP: 10(4)	30	27(NA)	66.5 (4.8)	*P* < 0.05	NINDS-SPSP	NA: 10	1.5	3	8	2
	HC: 9(4)	–	NA	66.6 (4.8)	(FDR)						
Cordato et al., 2005	PSP: 21(7)	23.1	25.4(3.2)	70.3(6.4)	*P* < 0.05	NINDS-SPSP	Definite PSP: 5	1.5	NA	8	9
	HC: 23(9)	–	29.4(0.9)	71.5(7.2)	(unclear)		NA: 16				
Padovani et al., 2006	PSP: 14(7)	22.1	25.8(2.7)	73.0 (5.6)	*P* < 0.005	NINDS-SPSP	Definite PSP: 14	1.5	1	8	22
	HC: 14(7)	–	NA	65.6 (4.1)	(FDR)						
Takahashi et al., 2011	PSP: 16(5)	NA	21.0(4.4)	64.9(6.4)	*P* < 0.001	NINDS-SPSP	Probable PSP: 16	1.5	5	8	8
	HC: 20(8)	–	29.8(0.6)	64.8 (6.9)	(uncorrected)						
Giordano et al., 2013	PSP: 15(7)	38.33	21.2(1.2)	68.9(1.2)	*P* < 0.05	NINDS-SPSP	Probable PSP: 15	3	1.2	8	11
	HC: 15(7)				(FWE)						
		–	29.8(0.6)	65.5(6.1)							

*PSP, progressive supranuclear palsy; F, female; UPDRS, Unified Parkinson's Disease Rating Scale; MMSE, mini-mental state examination; SD, standard deviation; T, tesla; FWHM, full-width at half maximum; #, number; FWE, family-wise error; FDR, false discovery rate; NA, not available; NINDS-SPSP, National Institute of Neurological Disorders and Stroke (NINDS) and the Society for PSP*.

### PD < HC

Thirty-two studies were identified in the meta-analysis (Figure 1) and a total of 24 studies on PD were included (Burton et al., 2004; Cordato et al., 2005; Nagano-Saito et al., 2005; Beyer et al., 2007; Ramirez-Ruiz et al., 2007; Feldmann et al., 2008; Karagulle Kendi et al., 2008; Camicioli et al., 2009; Jubault et al., 2009; Martin et al., 2009; Pereira et al., 2009; Sanchez-Castaneda et al., 2009; Tir et al., 2009; Dalaker et al., 2010; Kostic et al., 2010; Lee et al., 2010; Cerasa et al., 2011; Focke et al., 2011; Meppelink et al., 2011; Compta et al., 2012; Fernández-Seara et al., 2012; Hong et al., 2012; Ibarretxe-Bilbao et al., 2012; Tessitore et al., 2012). Seven hundred and sixteen PD patients and 535 HC subjects met the inclusion criteria for meta-analysis. The main characteristics of these included populations are presented in Table 2. Among these participants, 60 patients from three studies had mild cognitive impairment (Beyer et al., 2007; Dalaker et al., 2010; Lee et al., 2010) and 16 patients from one study had dementia (Sanchez-Castaneda et al., 2009). A total of 117 foci presented with decreased GMV were reported. ALE meta-analysis identified reduced GMV in the following: (1) frontal lobe including the bilateral middle, inferior frontal gyrus, and left precentral gyrus, (2) parietal lobe including bilateral precuneus and right superior parietal lobule, and (3) left middle temporal gyrus and right cuneus (Figure 2). No increased regions relative to HC were found in PD patients. Similar findings were obtained after excluding the aforementioned four studies conducted on PD patients with cognitive impairment (Supplemental Figure 1).

**Table 2:** **Summary of 24 VBM studies included for meta-analysis in PD**.

**Study**	**Number of subjects (*F*)**	**UPDRS-III**	**MMSE (*SD*)**	**Age (*SD*)**	**Significance threshold**	**Diagnostic criteria**	**Scanner (*T*)**	**Thickness (mm)**	**FWHM (mm)**	**# Foci**
Dalaker et al., 2010	PD: 42(17)	24.4	28.9(1.2)	64.6 (9.6)	*P* < 0.05	Gelb et al., 1999	1.5	1	8	5
	HC: 37(15)	–	28.9(1.2)	63.9 (9.5)	(FDR)					
Martin et al., 2009	PD: 26(9)	15.8	NA	59.8 (7.7)	*P* < 0.05	Calne et al., 1992	1.5	1	12	0
	HC: 14(6)	–	NA	56.8 (7.8)	(FDR)					
Pereira et al., 2009	PD: 36(14)	26.6	27.9(2.1)	73.3 (5.6)	*P* < 0.05	Daniel and Lees, 1993	1.5	1.5	12	30
	HC: 20(10)	–	28.7(1.8)	72.7 (6.7)	(FDR)					
Tir et al., 2009	PD: 19(8)	21	NA	61.6 (7.6)	*P* < 0.05	Hughes et al., 1992	1.5	1.3	12	2
	HC: 14(9)	–	NA	59.2 (7.6)	(unclear)					
Sanchez-Castaneda et al., 2009	PD: 16(5)	35.5	21.8(4.1)	71.1 (7.2)	*P* < 0.05	NA	1.5	1.3	8	2
	HC: 16(8)	–	28.6(2.0)	71.8 (7.6)	(FWE)					
Ramirez-Ruiz et al., 2007	PD: 38(23)	26.8	28.1(NA)	NA	*P* < 0.001	Hughes et al., 1992	1.5	1.5	12	12
	HC: 21(12)	–	29.4(2.3)	NA	(uncorrected)					
Jubault et al., 2009	PD: 23(10)	29.1	NA	64.0 (5.5)	*P* < 0.05	Hughes et al., 1992	3	1	12	0
	HC: 18(10)	–	NA	62.2 (5.4)	(FDR)					
Feldmann et al., 2008	PD: 50(20)	34.1	27.0(1.7)	61.6 (7.5)	*P* < 0.05	NA	1.0	2	8	0
	HC: 16(7)	–	28.7(0.7)	54.3 (10.4)	(FDR)					
Cerasa et al., 2011	PD: 72(31)	21.8	≥26	61.2(NA)	*P* < 0.05	Hughes et al., 1992	1.5	1.2	8	0
	HC: 32(15)	–	NA	65.1(8.2)	(FWE)					
Compta et al., 2012	PD: 18(6)	28.5	28(NA)	69(NA)	*P* < 0.05	Hughes et al., 1992	3	1	8	5
	HC: 12(8)	–	30(NA)	71.5(NA)	(FWE)					
Karagulle Kendi et al., 2008	PD: 12(7)	NA	28.2(NA)	62.1(12.7)	*P* < 0.05	Gelb et al., 1999	3	2	10	0
	HC: 13(5)	–	28.8(NA)	58.0(7.3)	(unclear)					
Nagano-Saito et al., 2005	PD: 39(NA)	25.5	28.4(1.9)	61.8(8.1)	*P* < 0.05	Calne et al., 1992	1.5	1.3	8	0
	HC: 31(NA)	–	29.2(1.2)	63.5(8.8)	(unclear)					
Hong et al., 2012	PD: 35(18)	16.3	28.4(NA)	64.5(NA)	*P* < 0.001	Hughes et al., 1992	3	1.2	6	11
	HC: 25(NA)	–	≥28	65.4(NA)	(uncorrected)					
Cordato et al., 2005	PD: 17(4)	23.1	28.6(1.2)	67.7(6.7)	*P* < 0.05	Hughes et al., 1992	1.5	1	8	1
	HC: 23(9)		29.4(0.9)	71.5(7.2)	(unclear)					
Tessitore et al., 2012	PD: 12(NA)	NA	28.3(2.2)	NA	*P* < 0.05	Hughes et al., 1992	3	1.2	8	2
	HC: 12(NA)		NA	NA	(FWE)					
Ibarretxe-Bilbao et al., 2012	PD: 16(4)	15.4	29.6(0.5)	55.9(8.1)	*P* < 0.05	Hughes et al., 1992	3	1	15	0
	HC: 15(3)		29.9(0.3)	57.7(9.5)	(FWE)					
Meppelink et al., 2011	PD: 24(NA)	NA	≥24	NA	*P* < 0.05	Hughes et al., 1992	3	1	10	16
	HC: 14(NA)		NA	NA	(FDR)					
Burton et al., 2004	PD: 31(8)	25.8 1.2	26.4(1.9)	75.2(5.2)	*P* < 0.01	Hughes et al., 1992	1.5	1.6	8	8
	HC: 36(16)		28.1(1.6)	75.1(6.6)	(unclear)					
Beyer et al., 2007	PD: 20(11)	NA	28.2(2.1)	72.5(8.5)	*P* < 0.05	Larsen et al., 1994	1.5	1.6	8	0
	HC: 20(10)		29.6(0.7)	73.3(6.3)	(FWE)					
Kostic et al., 2010	PD: 40(19)	20.6	27.6(NA)	66(NA)	*P* < 0.001	Hughes et al., 1992	1.5	1.5	8	4
	HC: 26(12)		NA	63(NA)	(uncorrected)					
Lee et al., 2010	PD: 41(20)	19.9	25.4(3.4)	71.3(6.3)	*P* < 0.001	Hughes et al., 1992	3	1.2	6	3
	HC: 21(NA)		≥28	70.7(2.7)	(uncorrected)					
Focke et al., 2011	PD: 21(6)	NA	NA	65.2(8.0)	*P* < 0.001	Hughes et al., 1992	3	1.5	8	2
	HC: 22(9)		NA	69.3(9.1)	(uncorrected)					
Fernández-Seara et al., 2012	PD: 25(18)	12.5	27.9(2.3)	63.2(6.6)	*P* < 0.05	Hughes et al., 1992	3	1	8	11
	HC: 34(12)		NA	63.5(6.6)	(unclear)					
Camicioli et al., 2009	PD: 43(19)	16.71.9	28.2(1.7)	70.7(4.0)	*P* < 0.01	Hughes et al., 1992	1.5	1.5	12	3
	HC: 43(19)		28.4(1.6)	71.0(4.5)	(FDR)					

PD, Parkinson’s disease; F, female; UPDRS, Unified Parkinson’s Disease Rating Scale; MMSE, mini-mental state examination; SD, standard deviation; T, tesla; FWHM, full-width at half maximum; #, number; FDR, false discovery rate; FWE, family-wise error; NA, not available.

### PSP < PD

Given that only two of three studies (Cordato et al., 2005; Focke et al., 2011; Giordano et al., 2013) directly investigated GMV differences between PSP and PD using the whole brain coordinate (Focke et al., 2011; Giordano et al., 2013), meta-analysis was not highly reliable. Therefore, ALE subtraction analysis was performed to compare GM differences between PSP and PD (Eickhoff et al., 2011). The subtraction analysis identified decreased GMV in the bilateral midbrain, thalamus, and insula in PSP compared with PD (Table 3 and Figure 2). The reverse contrast did not show any regions of GM atrophy. Similar findings were found using subtraction analysis between PSP (non-MCI) and PD (non-MCI) studies without including patients with mild cognitive impairment or dementia (Supplemental Figure 1).

**Table 3:** **Changes in gray matter volume**.

**Cluster No**.	**Volume (mm^3^)**	**Talairach coordinates (weighted center)**			**Macroanatomical and cytoarchitectonic region**	**Contributors**
		***X***	***Y***	***Z***		
**PSP < HC**						
1	4008	0.19	−23.61	6.47	Thalamus (th-temporal, th-prefrontal; left and right lobe); red nucleus (left and right lobe)	Cordato et al., 2005; Boxer et al., 2006; Padovani et al., 2006; Agosta et al., 2010; Lehericy et al., 2010
2	2048	−39.31	7.98	10.27	Insula (left lobe); inferior frontal gyrus (BA44, left lobe); precentral gyrus (left lobe)	Brenneis et al., 2004; Cordato et al., 2005; Boxer et al., 2006; Padovani et al., 2006; Agosta et al., 2010; Takahashi et al., 2011
3	1296	38.3	14.83	11.13	Insula (BA13, right lobe); inferior frontal gyrus (BA44, right lobe)	Brenneis et al., 2004; Boxer et al., 2006; Padovani et al., 2006; Ghosh et al., 2012
4	328	37.01	−4.24	10.12	Insula (BA13, right lobe); claustrum (right lobe)	Agosta et al., 2010; Ghosh et al., 2012
5	216	−10.31	1.04	16.78	Caudate nucleus (left lobe)	Cordato et al., 2005; Agosta et al., 2010
6	160	−1.91	44.47	6.52	Anterior cingulate cortex (left lobe)	Brenneis et al., 2004; Agosta et al., 2010
**PD < HC**						
1	608	−28.61	−10.08	58.63	Middle frontal gyrus (BA6, left lobe)	Fernández-Seara et al., 2012; Hong et al., 2012
2	448	42.89	36.64	11.38	Inferior frontal gyrus (BA46; right lobe)	Burton et al., 2004; Pereira et al., 2009
3	432	−27.02	−76.51	36.43	Precuneus (BA19; left lobe)	Ramirez-Ruiz et al., 2007; Meppelink et al., 2011; Hong et al., 2012
4	360	−42.82	19.14	−12.1	Inferior frontal gyrus (BA47; left lobe)	Ramirez-Ruiz et al., 2007; Pereira et al., 2009; Meppelink et al., 2011
5	304	−42.45	6.47	39.69	Middle frontal gyrus (left lobe)	Pereira et al., 2009; Kostic et al., 2010
6	272	31.77	31.64	31.7	Middle frontal gyrus (right lobe)	Burton et al., 2004; Ramirez-Ruiz et al., 2007
7	264	−13.58	−29.91	67.51	Precentral gyrus (BA 6,4a; left lobe)	Fernández-Seara et al., 2012
8	256	4.07	−71.39	22.06	Precuneus (right lobe); cuneus (right lobe)	Lee et al., 2010; Compta et al., 2012
9	248	16.78	−52.38	62.58	Superior parietal lobule (5L, 7PC, 5M; right lobe)	Pereira et al., 2009; Meppelink et al., 2011
10	240	−57.21	−44.53	−12.42	Middle temporal gyrus (left lobe)	Pereira et al., 2009; Hong et al., 2012
11	200	46.04	20.42	0.93	Inferior frontal gyrus (BA47; right lobe); superior parietal lobule (7A; right lobe)	Burton et al., 2004; Pereira et al., 2009
12	200	0.32	−64.31	47.8	Precuneus (BA7; left lobe)	Pereira et al., 2009; Compta et al., 2012
13	120	32.13	31.85	−3.55	Inferior frontal gyrus (BA47; right lobe)	Dalaker et al., 2010; Meppelink et al., 2011
**PSP < PD**						
1	3928	−0.09	−23.36	6.47	Thalamus (th-temporal, th-prefrontal; left and right lobe); red nucleus (left and right lobe)	Cordato et al., 2005; Boxer et al., 2006; Padovani et al., 2006; Agosta et al., 2010; Lehericy et al., 2010
2	1608	−37.63	9.03	10.24	Insula (BA13; left lobe)	Brenneis et al., 2004; Cordato et al., 2005; Boxer et al., 2006; Padovani et al., 2006; Agosta et al., 2010; Takahashi et al., 2011
3	280	37.26	17.33	10.97	Insula (BA13; right lobe)	Padovani et al., 2006

*No., number; PSP, progressive supranuclear palsy; HC, healthy control; PD, Parkinson's disease; th-, thalamus; BA, Brodmann area*.

## Discussion

Quantitative meta-analysis of VBM studies on PSP showed decreased GMV in the thalamus, midbrain, basal ganglia, insula, and frontal lobe, whereas quantitative meta-analysis of VBM studies on PD revealed reduced GMV in the frontal lobe, parietal lobe, left middle temporal gyrus, and right cuneus. In addition, subtraction analysis showed GM atrophy in the thalamus, midbrain, and insula lobe in PSP compared with PD.

In this study, reduced GMV in PD was observed in the following: (1) frontal lobe including bilateral middle, inferior frontal gyrus, and left precentral gyrus, (2) parietal lobe including bilateral precuneus and right superior parietal lobule, and (3) left middle temporal gyrus and right cuneus. The previous results showed changes in PD localized in the left inferior frontal gyrus, left superior temporal gyrus, and left insula lobe (Pan et al., 2012). The main results are consistent. The differences between the present findings and those of previous VBM studies on PD may be the result of the difference in methods (“signed differential mapping” method was used in our previous study and ALE method was used in the present study) and number of participants (498 PD patients and 375 HC were included in our previous study, whereas 716 PD patients and 535 HC were included in the present study).

We found decreased GMV in the frontal lobe, including inferior frontal gyrus and precentral gyrus in both PSP and PD. These results are supported by some fMRI and pathological studies (Albers et al., 2000; Whitwell et al., 2011; Kehagia et al., 2012; Yang et al., 2013). The precentral gyrus belongs to the motor cortex and is the origin of the pyramidal tract (Bonelli and Cummings, 2007). Additionally, the inferior frontal gyrus (Brodmann area 44), affects movement control, including speech and hand actions (Rizzolatti et al., 2002; Bonelli and Cummings, 2007). It also participates in response inhibition (Picton et al., 2007; Forstmann et al., 2008). Therefore, the impairment of the precentral gyrus and inferior frontal gyrus may partially contribute to the motor and non-motor deficits of both disorders.

Interestingly, GM atrophy in the bilateral midbrain, thalamus, and insula was found to be more severe in PSP than in PD, although no significant foci were revealed in these structures in the meta-analysis of VBM studies on PD. The pathological hallmarks of PSP include globose neurofibrillary tangles, tufted tau-positive astrocytes, and neuronal loss in the midbrain (Dickson et al., 2007; Williams and Lees, 2009). The midbrain not only maintains equilibrium (Barsottini et al., 2010), but also has a role in vertical saccades (Da Cunha et al., 2002; Kato et al., 2003). More severe atrophy of the midbrain can contribute to disturbances in balance and vertical gaze movement in PSP. Although dopaminergic neuron loss in the substantia nigra in PD was found to be the main pathology in PD (Halliday et al., 1996), we failed to find midbrain atrophy in PD by means of VBM meta-analysis. Therefore, severe atrophy of the midbrain could be a neuroanatomical marker that can be used to differentiate PSP from PD.

Subtraction analysis showed that GMV reductions in the bilateral thalamus are more severe in PSP patients compared with PD patients. Atrophy of the thalamus revealed in PSP in the current meta-analysis of VBM studies is consistent with the findings of studies using PET (Juh et al., 2005) and single-photon emission computed tomography (Kimura et al., 2011). Pathological research on PSP also confirmed that the thalamus often exhibits some degree of neuronal loss and gliosis, particularly in the lateral and ventral anterior thalamus nuclei (Dickson et al., 2007). Postural instability in PSP patients has been associated with impairment of the thalamus (Zwergal et al., 2011), which has a significant role in motor functions through both functional and structural connections with the basal ganglia and motor regions of the frontal lobe (Herrero et al., 2002; Cordato et al., 2005; Bonelli and Cummings, 2007). Although altered thalamic neuronal activity in the cortical-striatal-thalamic loop is the main pathophysiology in PD (Halliday, 2009), the present study found no obvious thalamus atrophy in PD, confirming that thalamus impairment in PD consists of functional deficits other than structural changes. Our main findings stayed consistent after excluding PSP or PD patients with cognitive impairment, we can not, suggest that the thalamus may not be closely related to the cognitive impairment of PD or PSP patients since these PSP or PD patients with cognitive impairment accounted for a small portion of our patient samples. However, future studies on PSP or more advanced PD patients will increase current understanding on the impairment of the thalamus.

Interestingly, subtracting analysis showed that insula atrophy was more severe in PSP than in PD. Very few articles have investigated the insula impairment of PSP. A previous PET study found hypometabolism in the insula of PSP patients (Hirano et al., 2010). Other studies have found that dysfunction of the insula could lead to abnormalities in articulation and emotions (Augustine, 1996; Baldo et al., 2011). Although some neuroimaging studies using structural MRI, fMRI, or PET (Kikuchi et al., 2001; Stefurak et al., 2003; Pinto et al., 2004; Pavese et al., 2010) have reported insula damage in PD patients, these patients exhibited fatigue or dysarthria. Most included studies on PD did not provide detailed information on motor and non-motor symptoms, such as severity of dysarthria, static tremor, or fatigue, which may partially explain why insula atrophy was not proved in this study.

However, the finding of subtraction analysis in the current study was different from the results of two VBM studies (Focke et al., 2011; Giordano et al., 2013) which directly compared GMV on PSP and PD, which found the atrophy was more severe in cerebellum, parahippocampal gyrus, middle frontal gyrus, occipital pole, and precentral gyrus in PSP than PD. However, the sample size of these two studies was relatively small. Future large sample size comparison studies on PSP–PD with multimodal imaging techniques will help to answer the difference. This study has several limitations, which could have biased our results. First, unpublished studies and studies with insufficient data were excluded, and the studies were limited to those published in English. Second, the voxel-wise meta-analyses were based on the reported stereotactic coordinates with significant differences rather than on raw data, and the included patients from different studies may have experienced various severities of disease or therapy, thereby potentially influencing the results. Third, the heterogeneity of the methodologies used in VBM studies, including different expression of GM changes (modulated GMV and unmodulated GMD), FWHM and statistical thresholding methods, could not be ruled out entirely. Additionally, PSP is classified into Richardson syndrome and PSP-Parkinsonism (PSP-P) according to different clinical signs (Williams et al., 2005). Given that only two articles (Agosta et al., 2010; Lehericy et al., 2010) mention the phenotypes, we could not perform separate meta-analysis of each phenotype or between PSP-P and PD.

In summary, the present meta-analysis indicated that both PSP and PD patients suffer from GM atrophy in the precentral gyrus and inferior frontal gyrus. GM atrophy was also identified in the bilateral midbrain, thalamus, and insula in PSP patients compared with PD patients, and could thus be an anatomical biomarker that can be used to differentiate these two disorders. Future studies with larger samples of patients in the early stage of disease, as well as multimodal methods, may better expound the changes.

### Conflict of interest statement

The authors declare that the research was conducted in the absence of any commercial or financial relationships that could be construed as a potential conflict of interest.
