# Supplementary material for: Voxelwise meta-analysis of gray matter anomalies in progressive supranuclear palsy and Parkinson's disease using anatomic likelihood estimation
Source: Front Hum Neurosci. 2014 Feb 18;8:63. doi: 10.3389/fnhum.2014.00063 (PMC3927227; doi:10.3389/fnhum.2014.00063)
Supplement: Supplementary file 1 [file Presentation1.PDF]

Supplementary material:

PSP (non-MCI) < HC

We included nine PSP – HC articles in the present study and thirty PSP patients had mild cognitive impairment (MCI) from two articles (Padovani et al., 2006, Takahashi et al., 2011). Therefore, comparison between PSP patients without MCI and healthy controls were conducted. Seven articles (Brenneis et al., 2004, Cordato et al., 2005, Boxer et al., 2006, Agosta et al., 2010, Lehericy et al., 2010, Ghosh et al., 2012, Giordano et al., 2013) including 115 non-MCI PSP patients and 183 HC were conducted. Ginger ALE (http://brainmap.org/ale/index.heml) was used to transform reported coordinates and did the meta-analysis. The results were finally threshold and then corrected for multiple comparisons using False Discovery Rate (FDR) at $p<0.05$ with a minimum cluster size of 100 mm^3. GM reduction was identified in the bilateral insula, cerebellum, left thalamus, caudate body, and anterior cingulate by using the ALE meta-analysis method (Supplemental Figure 1).

PD (non-MCI) < HC

Twenty-four PD – HC articles were included in the present study, 60 patients from three studies had mild cognitive impairment (Beyer et al., 2007, Dalaker et al., 2010, Lee et al., 2010) and 16 patients from one study had dementia (Sanchez-Castaneda et al., 2009). Moreover, two articles (Beyer et al., 2007, Dalaker et al., 2010) of them didn't report the coordinates of PD with MCI separately. Therefore, we deleted these four articles and re-analyzed the data. A total of 20 studies (Burton et al., 2004,

Cordato et al., 2005, Nagano-Saito et al., 2005, Ramirez-Ruiz et al., 2007, Feldmann et al., 2008, Karagulle Kendi et al., 2008, Camicioli et al., 2009, Jubault et al., 2009, Martin et al., 2009, Pereira et al., 2009, Tir et al., 2009, Kostic et al., 2010, Cerasa et al., 2011, Focke et al., 2011, Meppelink et al., 2011, Compta et al., 2012, Fernandez-Seara et al., 2012, Hong et al., 2012, Ibarretxe-Bilbao et al., 2012, Tessitore et al., 2012) including 597 PD patients without cognitive impairment and 441 HC were included. Ginger ALE (http://brainmap.org/ale/index.heml) was used to transform reported coordinates and did the meta-analysis. The results were finally threshold and then corrected for multiple comparisons using False Discovery Rate (FDR) at $p<0.05$ with a minimum cluster size of 100 mm^3. In PD patients without cognitive impairment, ALE meta-analysis identified reduced GMV in the frontal lobe including bilateral middle and inferior frontal gyrus and left precentral gyrus, parietal lobe including left precuneus and right superior parietal lobule, left middle temporal gyrus (Supplemental Figure 1).

Subtraction meta-analysis between PSP (non-MCI) and PD (non-MCI)

A subtraction meta-analysis was performed in order to compare GM differences in PSP and PD patients without cognitive impairment by using Ginger ALE method (http://brainmap.org/ale/index.heml). The subtraction meta-analysis found that decreased GMV in the left midbrain, left caudate body, left thalamus and bilateral insula in PSP when compared with PD. The reverse contrast did not show any regions of GM atrophy (Supplemental Figure 1).

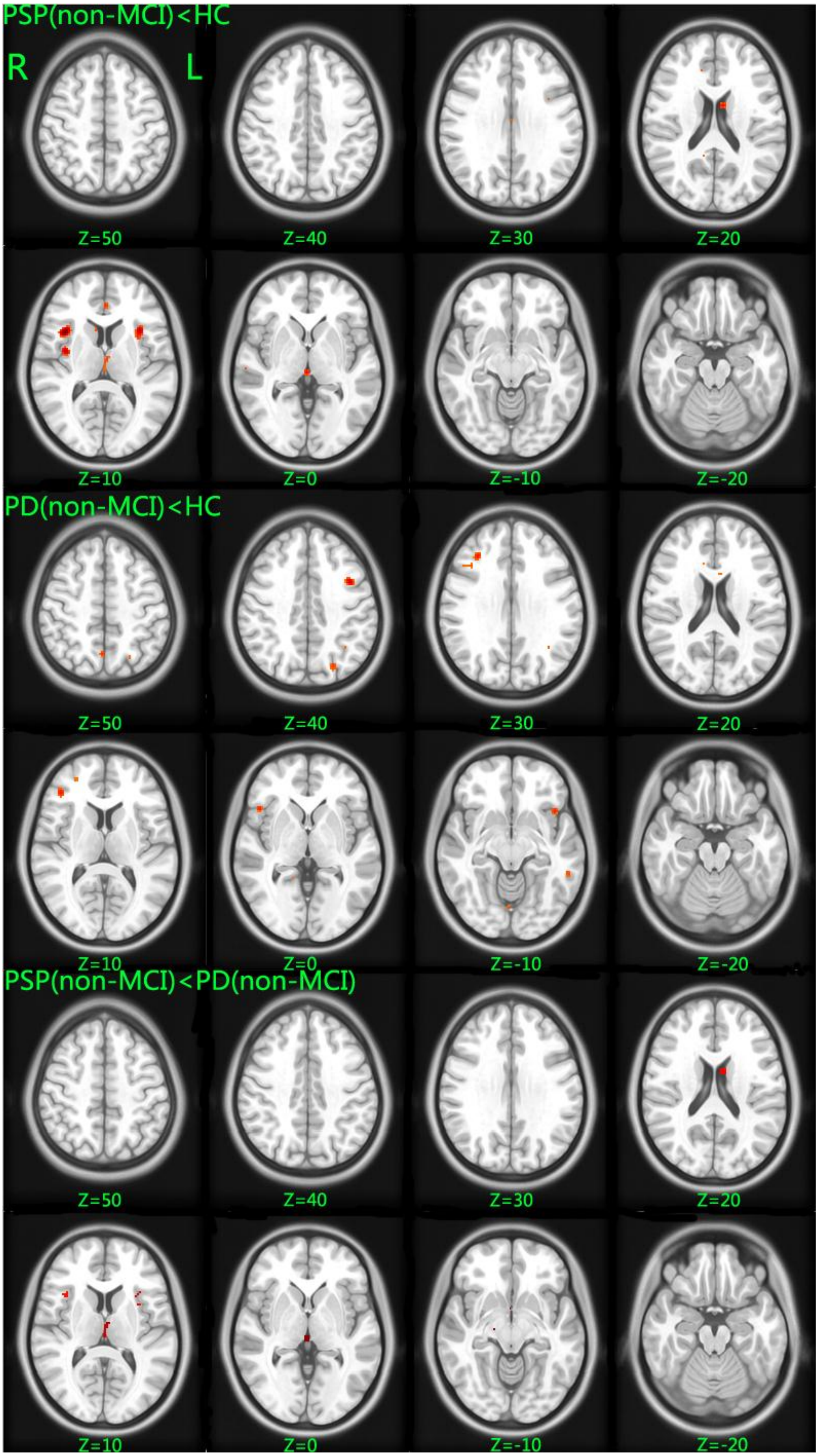
PSP(non-MCI)<HC
R
L
Z=50
Z=40
Z=30
Z=20
Z=10
Z=0
Z=-10
Z=-20
PD(non-MCI)<HC
Z=50
Z=40
Z=30
Z=20
Z=10
Z=0
Z=-10
Z=-20
PSP(non-MCI)<PD(non-MCI)
Z=50
Z=40
Z=30
Z=20
Z=10
Z=0
Z=-10
Z=-20

Supplemental Figure 1
